# Non-invasive High-Frequency Oscillatory Ventilation as Initial Respiratory Support for Preterm Infants With Respiratory Distress Syndrome

**DOI:** 10.3389/fped.2021.792160

**Published:** 2022-01-11

**Authors:** Shu-Hua Lai, Ying-Ling Xie, Zhi-Qing Chen, Rong Chen, Wen-Hong Cai, Luo-Cheng Wu, Yun-Feng Lin, Yi-Rong Zheng

**Affiliations:** ^1^Department of Neonatology, Fujian Maternity and Child Health Hospital, Affiliated Hospital of Fujian Medical University, Fuzhou, China; ^2^Fujian Key Laboratory of Women and Children's Critical Diseases Research, Fujian Maternity and Child Health Hospital, Fuzhou, China; ^3^Fujian Branch of Shanghai Children's Medical Center, Fuzhou, China; ^4^Fujian Children's Hospital, Fuzhou, China

**Keywords:** non-invasive high-frequency oscillatory ventilation, biphasic positive airway pressure, preterm infants, respiratory distress syndrome, non-invasive ventilation

## Abstract

**Objectives:** The aim of this study was to investigate the safety and feasibility of nHFOV as initial respiratory support in preterm infants with RDS.

**Methods:** This study retrospectively analyzed the clinical data of 244 premature infants with RDS who were treated in our hospital from January 2016 to January 2019 and divided into the nHFOV group (*n* = 115) and the BiPAP group (*n* = 129) based on the initial respiratory support method.

**Results:** Respiratory outcomes showed that the rate of NIV failure during the first 72 hours of life in the nHFOV group was significantly lower than that in the BiPAP group. The time of NIV in the nHFOV group was significantly shorter than that in the BiPAP group. The time of supplemental oxygen in the nHFOV group was significantly shorter than that in the BiPAP group. The incidence of air leakage syndrome in the nHFOV group was significantly lower than that in the BiPAP group, and the length of hospital stay of the nHFOV group was also significantly shorter than that in the BiPAP group. Although the rate of infants diagnosed with BPD was similar between the two groups, the rate of severe BPD in the nHFOV group was significantly lower than that in the BiPAP group.

**Conclusion:** This study showed that nHFOV as initial respiratory support for preterm infants with RDS was feasible and safe compared to BiPAP. Furthermore, nHFOV can reduce the need for IMV and reduce the incidence of severe BPD and air leak syndrome.

## Introduction

Respiratory distress syndrome (RDS) is one of the most common complications in preterm infants and the most common reason for premature death. A large proportion of preterm infants with RDS require invasive mechanical ventilation (IMV) at an early stage of life. Although ventilation is usually life-saving, it can also cause many complications, such as air leak syndrome, lung injury, and neurodevelopmental impairment (1–3). Neonatologists are increasingly using non-invasive ventilation (NIV) in the neonatal intensive care unit (NICU) to reduce these adverse effects of IMV; among them, biphasic positive airway pressure (BiPAP) is the classic non-invasive ventilation mode. Reports have shown that NIV is feasible in clinical practice and is associated with reducing the need for intubation and decreasing ventilator-related lung injury and other complications (2, 4). Non-invasive high-frequency oscillatory ventilation (nHFOV) is a promising new mode of NIV that can reduce the risk and complications of IMV (5, 6). However, there are few reports on the application of nHFOV for the treatment of premature infants with RDS. We hypothesized that nHFOV was safe and effective as an initial respiratory support for preterm infants with RDS and had more advantages than BiPAP. We conducted a retrospective controlled study to evaluate the efficacy, safety and advantages of nHFOV for the treatment of premature infants with RDS.

## Methods

The present study was approved by the ethics committee of our hospital and adhered to the tenets of the Declaration of Helsinki. Additionally, all parents of the patients signed the consent form before participating in the study.

### Patients

**Figure 1:**
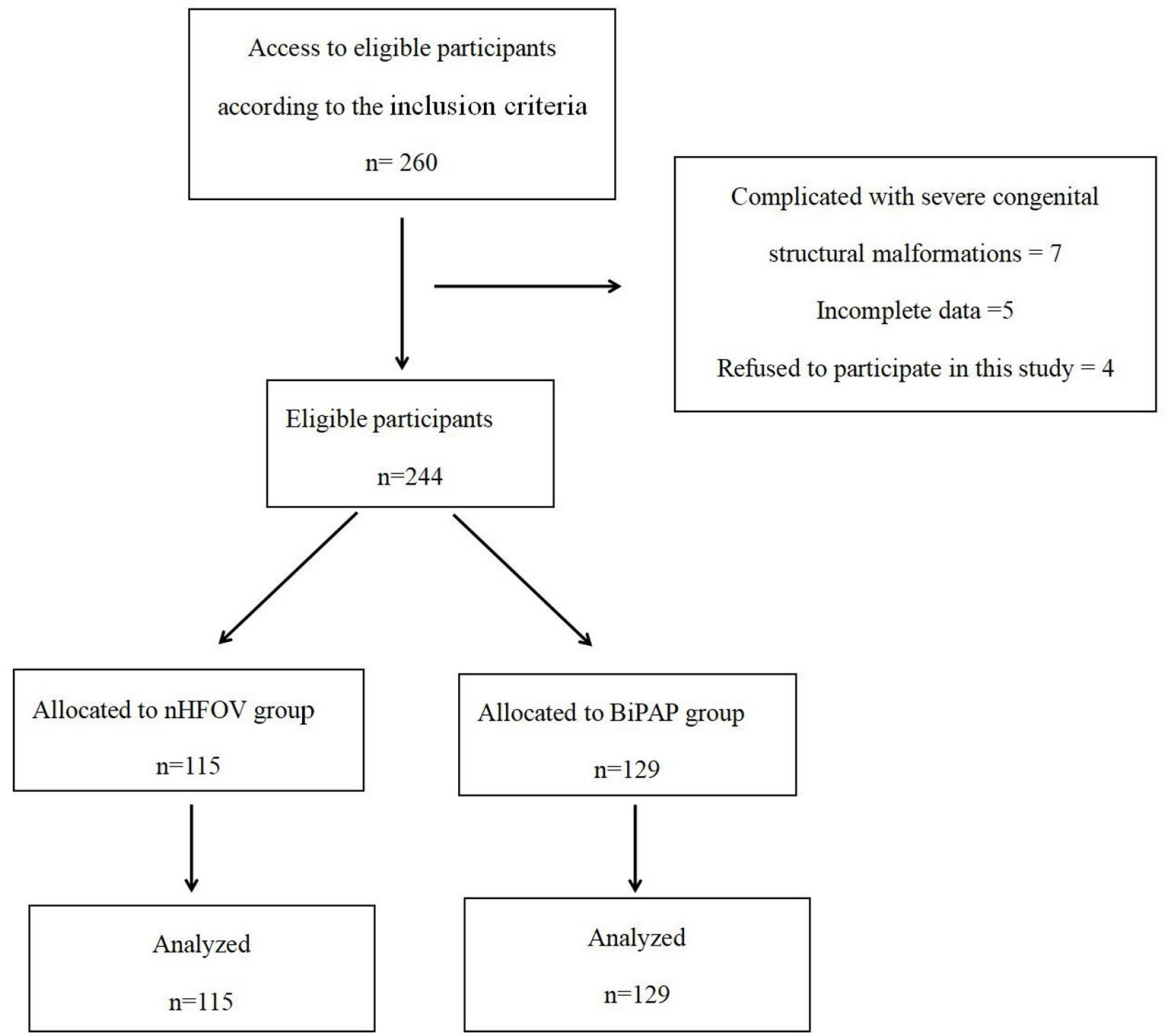
The patient flow diagram of the study.

Our hospital started using nHFOV in January 2018, and then RDS initial respiratory support was gradually transitioned from the previous BiPAP to nHFOV. Therefore, BiPAP respiratory support was also used in some patients from 2018 and 2019. From January 2016 to January 2019, there was no change in other treatment except respiratory support. This study retrospectively analyzed the clinical data of 244 premature infants with RDS who were treated in our hospital from January 2016 to January 2019 and were divided into two groups based on the initial respiratory support methods. The nHFOV group had 115 premature infants who received nHFOV as initial respiratory support, and the BiPAP group had 129 premature infants who received BiPAP as initial respiratory support (Figure 1). All the patients were definitely diagnosed with RDS based on the diagnostic criteria. The diagnostic criteria of RDS are as follows (6): (1) High-risk factors: maternal diabetes during pregnancy, intrauterine infection, premature delivery, premature rupture of membranes for more than 24 h, intrauterine distress, asphyxia during delivery, etc. (2) Clinical symptoms: progressive aggravation of tachypnea within 6 h after birth (>60 times/min); cyanosis, three depressions in inhalation and obvious expiratory moans, irregular breathing, and apnoea; and decreased respiratory sounds in both lungs were detected on auscultation. (3) Typical chest X-ray features.

The inclusion criteria were as follows: (1) premature infants with gestational age of 25–34 weeks; (2) diagnosed with RDS within 24 h of birth and received nHFVO or BiPAP as initial respiratory support. The exclusion criteria were as follows: (1) complications involving severe congenital structural malformations such as congenital heart disease, congenital diaphragmatic hernia, respiratory tract malformation, and severe digestive tract malformation; (2) incomplete data; and (3) the parents of the infants refused to participate in this study.

### Management in the Delivery Room

Drying, maintaining warmth and treating the umbilical cord immediately after birth were performed in the delivery room. Then, based on the breathing condition of the premature baby, we started respiratory support in the delivery room with nasal continuous positive airway pressure support or intermittent positive pressure ventilation after intubation. The initial management in the delivery room for all the babies in the two groups was the same.

### Respiratory Management

Daily care was performed as needed for preterm infants in the NICU, along with continuous monitoring of pulse oxygen saturation, respiratory rate, and heart rate. nHFOV or BiPAP was started within 24 h of life with clinical signs of respiratory distress. Short binasal prongs (Infant Flow, CareFusion, California, USA) were used as the interface for the two NIV devices. The orogastric tube was kept open to decompress the stomach and to facilitate feeding. All of the preterm infants were given prophylactic caffeine on the first day of life. Caffeine was administered at a loading dose of 20 mg/kg caffeine citrate, and then a daily maintenance dose of 5–10 mg/kg coffee citrate was given. Our unit did not implement preventive use of surfactants. Surfactant requirements were assessed for all preterm infants. Porcine surfactant (Curosurf, Chiesi Farmaceutici, Parma, Italy) was administered as a rescue therapy *via* the intubation, surfactant therapy, extubation (INSURE) method if the infant required ≥ 0.40 fraction of inspired oxygen (FiO2) to maintain the target oxygen saturation level of 90–95%. The first dose of surfactant therapy was 200 mg/kg. Additional surfactant doses of 100 mg/kg were administered if the infants still required ≥ 0.40 FiO2 to maintain the target oxygen saturation.

In the nHFOV group, a neonatal non-invasive high-frequency ventilator from Medin CNO (Medical Innovations GmbH, Puchheim, Germany) with an output oxygen concentration range of 21 to 100% was used. The initial parameters were as follows: average airway pressure (MAP) was 8 cmH_2_O, frequency was 9 Hz, amplitude was adjusted to achieve sufficient chest oscillation at rest, and FiO2 was 0.4. Based on blood gas analysis and transcutaneous oxygen saturation (SpO2) adjustment parameters, FiO2 was adjusted by 0.05, MAP was adjusted by 1 cmH_2_O, and frequency was adjusted by 1 Hz each time.

For infants in the BiPAP group, a baseline PEEP of 5 cmH_2_O was established, with a high PEEP of 9 cmH_2_O, a respiratory rate of 30/min, and an initial inspiratory time of 0.5 s (CareFusion, California, USA). The baseline PEEP was adjusted to 4–7 cmH_2_O, and the high PEEP was adjusted to 7–10 cmH_2_O.

If the infant remained clinically stable at minimum respiratory parameters with nHFOV (MAP: 6 cmH2O; FiO2: 0.30) and BiPAP (cycle rate: 15 times/min, lower CPAP: 3 cmH_2_O; higher CPAP: 5 cmH_2_O; FiO2: 0.30), good respiratory effort and maintenance of an oxygen saturation level of 90–95%, conditions were switched to a heated humidified high-flow nasal cannula or nasal continuous positive airway pressure (NCPAP). If the infant under NCPAP had a CPAP level of 3 cmH_2_O and FiO2 <0.25 and tolerated the treatment well for at least 24 h with no evidence of apnoea, the infant was weaned to supplemental oxygen or room air.

Failure of NIV was defined as at least one of the following (7): severe respiratory acidosis (pH ≤ 7.20 and PaCO2 ≥ 60 mmHg); hypoxemia (PaO_2_ ≤ 50 mmHg and FiO2 ≥ 0.6); recurrent apnoea associated with bradycardia ≥ 3 times/hour; or a single episode of apnoea that required bag-and-mask ventilation, pneumothorax or intestinal perforation, severe respiratory distress, or pulmonary hemorrhage.

The definition of bronchopulmonary dysplasia (BPD) was any oxygen dependence (FiO_2_ > 21%) of the newborn lasting more than 28 days. Based on the judgement of oxygen dependence and different types of respiratory support measures, BPD can be categorized into three degrees: mild, moderate and severe. Preterm infants with a gestational age less than 32 weeks were assessed at 36 weeks postmenstrual or discharge, and preterm infants with a gestational age more than 32 weeks were assessed at 56 days after birth or discharge. BPD was considered (1) mild for patients without oxygen inhalation; (2) moderate for those requiring oxygen inhalation and FiO2 was <30%; and (3) severe for those requiring oxygen inhalation, FiO2 was ≥ 30% or positive pressure ventilation was needed (8).

### Statistical Analysis

SPSS 25.0 was used for statistical analysis. Continuous data are presented as the mean ± standard deviation and range. With all continuous data, the normality of the distribution was tested, and they followed a normal distribution. Clinical parameters between the two groups were compared with independent samples *t* tests. χ2 tests were used to categorize the variables. A *p* value of < 0.05 was defined as significant.

## Results

The general data, birth situation and perinatal data of the participants in the two groups are shown in Table 1; there were no statistically significant differences between the two groups, which indicated that the baseline characteristics between the two groups were similar (Table 1).

**Table 1:** Comparison of general data between the two groups.

	**nHFOV group**	**BiPAP group**	***P*-value**
Number	115	129	
Gestational age (weeks)	29.1 ± 1.8	28.9 ± 1.6	0.457
Birthweight (kg)	1.1 ± 0.220	1.2 ± 0.242	0.198
Male/female	59/56	68/61	0.826
Multiple births	14 (12.1%)	17 (13.2%)	0.814
Apgar score at 5 min	8.2 ± 2.1	8.3 ± 2.0	0.979
Intra uterine growth retardation	21 (18.3%)	26 (20.2%)	0.708
Ante-natal steroids (incomplete and full course)	61 (53.0%)	78 (60.4%)	0.242
Cesarean section	55 (47.8%)	74 (57.4%)	0.136
Gestational diabetes mellitus	35 (30.4%)	46 (35.7%)	0.387
Pre-eclampsia	24 (20.9%)	29 (22.5%)	0.763
Maternal age (years)	29.2± 3.0	29.3 ± 2.5	0.744
Prolonged premature rupture of membranes > 18 h	26 (22.6%)	22 (17.1%)	0.276
Chorioamnionitis	13 (11.3%)	10 (7.8%)	0.343

*BiPAP, biphasic positive airway pressure; nHFOV, non-invasive high-frequency oscillatory ventilation*.

The respiratory outcomes showed that the rate of NIV failure in the first 72 h of life was significantly lower in the nHFOV group than in the BiPAP group (9 vs. 23, *P* = 0.021). The time of NIV in the nHFOV group was shorter than that in the BiPAP group (10.5 ± 4.3 vs. 12.7 ± 5.6, *P* = 0.032). Among infants with NIV failure, the time of first intubation in the BiPAP group was significantly earlier than that in the nHFOV group (2.0 ± 1.6 vs. 3.5 ± 2.3, *P* = 0.048), and the time of invasive ventilation in the nHFOV group was significantly shorter than that in the BiPAP group (3.2 ± 1.8 vs. 5.4 ± 2.0, *P* = 0.036). The time of supplemental oxygen in the nHFOV group was shorter than that in the BiPAP group (7.2 ± 4.8 vs. 8.5 ± 5.6, *P* = 0.040). The rate of the required surfactant was similar between the two groups (Table 2).

**Table 2:** Comparison of respiratory status between the two groups.

	**nHFOV group**	**BiPAP group**	***P*-value**
Number	115	129	
NIV failure in the first 72 hours of life	9 (7.8%)	23 (17.8%)	0.021
Required surfactant	46 (40.0%)	59 (45.7%)	0.366
Required ≥ 2 doses of surfactant	10 (8.7%)	17 (13.1%)	0.265
Time at first intubation (days)	3.5 ± 2.3	2.0 ± 1.6	0.048
Duration of NIV (days)	10.5 ± 4.3	12.7 ± 5.6	0.032
Duration of IMV (days)	3.2 ± 1.8	5.4 ± 2.0	0.036
Duration of supplemental oxygen (days)	7.2 ± 4.8	8.5 ± 5.6	0.040

*BiPAP, Biphasic positive airway pressure; nHFOV, non-invasive high-frequency oscillatory ventilation; IMV, invasive mechanical ventilation; NIV, Non-invasive ventilation*.

A comparison of the complications between the two groups showed that the incidence of air leakage syndrome in the nHFOV group was significantly lower than that in the BiPAP group (2 vs. 10, *P* = 0.038), and the length of hospital stay was also significantly shorter in the nHFOV group than in the BiPAP group (44.0 ± 10.9 vs. 47.3 ± 12.8, *P* = 0.033). Although the rate of infants diagnosed with BPD was similar between the two groups, the rate of severe BPD in the nHFOV group was significantly lower than that in the BiPAP group (1 vs. 7, *P* = 0.046). The incidence of nasal injury, retinopathy of prematurity requiring laser treatment, necrotizing enterocolitis, and mortality were similar between the two groups (Table 3).

**Table 3:** Comparison of complications between the two groups.

	**nHFOV group**	**BiPAP group**	***P*-value**
Number	115	129	
Nasal injury	5 (4.3%)	4 (3.1%)	0.738
Air leak syndrome	2 (1.7%)	10 (7.8%)	0.038
Retinopathy of prematurity required laser treatment	4 (3.5%)	9 (7.0%)	0.225
Necrotizing enterocolitis	8 (7.0%)	15 (11.6%)	0.213
BPD	17 (14.8%)	27 (20.9%)	0.212
Mild BPD	10 (8.7%)	11 (8.5%)	0.963
Moderate BPD	6 (5.2%)	9 (7.0%)	0.568
Severe BPD	1 (0.9%)	7 (5.4%)	0.046
Duration of hospitalization (days)	44.0± 10.9	47.3 ±12.8	0.033
Mortality	7 (6.1%)	8 (6.2%)	0.970

*BiPAP, biphasic positive airway pressure; nHFOV, non-invasive high-frequency oscillatory ventilation; BPD, bronchopulmonary dysplasia*.

## Discussion

Various forms of NIV are increasingly being used by neonatologists because of the potential adverse consequences of IMV, particularly ventilator-induced lung injury, and this restricted use of IMV in preterm infants might decrease lung inflammation and reduce the incidence of BPD and death (9). The most commonly used NIV method is BiPAP, and BiPAP is an effective therapy in the early management of RDS in preterm infants (10). nHFOV, as a novel mode of non-invasive ventilation, can improve the removal of carbon dioxide with the advantages of high-frequency ventilation and NIV (11).

Studies have shown that nHFOV can reduce the need for IMV compared to other NIV techniques (12–14). In this study, we found that compared to BiPAP, nHFOV as initial respiratory support reduced the need for IMV within the first 72 h of life. We also found that nHFOV decreased the rate of intubation, shortened the duration of IMV and postponed the first intubation. Compared with other forms of NIV, nHFOV retains a non-invasive interface and maintains continuous airway pressure, and it can also increase functional residual volume, maintain the opening of the upper airway, and prevent alveolar collapse, which can improve ventilation and oxygenation (15). Mukerji et al. used a lung model to compare nHFOV with other forms of NIV, and the results showed that nHFOV could remove carbon dioxide more effectively, promote alveolar revascularization and reduce the rate of tracheal intubation compared with other forms of NIV (16). We suggested that the higher NIV failure rate in the BiPAP group than in the nHFOV group might also be due to BiPAP offering synchronized nasal intermittent positive pressure ventilation using an abdominal capsule, which may be difficult to synchronize with infant breathing and does not result in larger tidal volumes (17). However, with nHFOV, there is no need for synchronization. nHFOV may improve ventilation by enhancing alveolar recruitment by applying higher MAP, and the functional residual capacity was increased (18). On the other hand, despite restricting the use of MAP on nHFOV to 10 cmH_2_O, this was still higher than BiPAP for the corresponding FiO_2_ levels. Binmanee et al. (19) proved that the use of high NIV (MAP ≥ 10 cmH_2_O) resulted in the avoidance of intubation in the majority of cases, without adverse effects. Yaser et al. (20) also found a higher MAP in the successful group than in the failure group as a prophylactic or rescue mode of NIV following extubation. Therefore, these differences may be due to the airway pressure itself rather than the pressure waveform generated from the two NIV modes.

The nHFOV group had a lower rate of air leak syndrome than the BiPAP group, and we suspected that this may be due to the small tidal volumes used, which results in less pressure-and-volume trauma from nHFOV (21). High-frequency oscillation ventilation is a type of oscillation with a high frequency of air flow. Through a diffusion mechanism, a small amount of gas was sent into or out of the airway ventilation method, and there was no synchronization and no man-machine confrontation (22). However, BiPAP offers synchronized nasal intermittent positive pressure ventilation using an abdominal capsule, which may be difficult to synchronize with infant breathing. This situation makes it very easy to create man-machine confrontation; even if the MAP was lower, air leak syndrome was more likely to occur.

BPD is a common and serious complication in premature infants (23). Studies have shown that IMV and high oxygen exposure are high-risk factors for BPD (24, 25). Although the rate of infants diagnosed with BPD was similar between the two groups, the rate of severe BPD in the nHFOV group was significantly lower than that in the BiPAP group. This difference may have resulted from the facilitation of gas exchange in neonates treated with nHFOV, and nHFOV was able to sustain oxygenation and ventilation while leading to improved alveolar or lung development (6, 26–28). These effects on lung development raise the possibility that nHFOV may reduce neonatal chronic lung disease. nHFOV reduces the duration of supplemental oxygen and the use of IMV, which can also help reduce the incidence of severe BPD.

nHFOV reduced the duration of supplemental oxygen and hospitalization, which may have benefitted from the reduction in airway inflammation, lung injury, and incidence of severe BPD by nHFOV. Of concern was the decrease in NEC and retinopathy of prematurity requiring laser treatment in thed nHFOV group, which may have been due to nHFOV reducing the duration of supplemental oxygen and IMV and the fluctuations in blood oxygen saturation, although there were no statistically significant differences.

Our study had some limitations, which may affect the validity of our findings. First, it was a retrospective study, and it was not a prospective randomized controlled study, and therefore, the study's objectivity was somewhat limited. Second, it was a single-center study with a small sample size. Third, the follow-up time was not long enough. Fourth, the study infants were not stratified by birth weight or gestational age, and there was a lack of infants at less than 25-weeks of gestation.

## Conclusion

This study showed that nHFOV as initial respiratory support in preterm infants with RDS was feasible and safe compared to BiPAP. Furthermore, nHFOV can reduce the need for IMV and reduce the incidence of severe BPD and air leak syndrome.

## Data Availability Statement

The data analyzed in this study is subject to the following licenses/restrictions: The data that support the findings of this study are available on request from the corresponding author. The data are not publicly available due to privacy or ethical restrictions. Requests to access these datasets should be directed to Shu-Hua Lai, laishuhua2014@163.com.

## Author Contributions

S-HL, Y-FL, and Y-RZ designed the study, collected the clinical data, performed the statistical analysis, participated in the operation, and drafted the manuscript. Y-LX , Z-QC, RC, W-HC, and L-CW participated in the operation and revised the article. All authors read and approved the final manuscript.

## Conflict of Interest

The authors declare that the research was conducted in the absence of any commercial or financial relationships that could be construed as a potential conflict of interest.

## Publisher's Note

All claims expressed in this article are solely those of the authors and do not necessarily represent those of their affiliated organizations, or those of the publisher, the editors and the reviewers. Any product that may be evaluated in this article, or claim that may be made by its manufacturer, is not guaranteed or endorsed by the publisher.

## Abbreviations

RDS: Respiratory distress syndrome
IMV: Invasive mechanical ventilation
NIV: Non-invasive ventilation
NICU: Neonatal intensive care unit
BiPAP: Biphasic positive airway pressure
nHFOV: Non-invasive high-frequency oscillatory ventilation
FiO2: Inspired oxygen
MAP: Average airway pressure
NCPAP: Nasal continuous positive airway pressure
BPD: Bronchopulmonary dysplasia.
